# Development of an Assessment Tool to Measure Healthy Eating in Navajo Children and Their Families^☆^

**DOI:** 10.1016/j.cdnut.2023.100074

**Published:** 2023-04-01

**Authors:** Shirley AA. Beresford, Eileen Rillamas-Sun, Kassia Rudd, Sonia K. Bishop, Desiree Deschenie, India J. Ornelas, Mark C. Bauer, Kevin A. Lombard

**Affiliations:** 1 Department of Epidemiology, School of Public Health, University of Washington, Seattle, WA, United States; 2 Division of Public Health Sciences, Fred Hutchinson Cancer Center, Seattle, WA, United States; 3 New Mexico State University Agricultural Science Center at Farmington, Farmington, NM, United States; 4 Department of Health Systems and Population Health, School of Public Health, University of Washington, Seattle, WA, United States; 5 Public Health, School of STEM (Science, Technology, Engineering and Mathematics), Diné College, Shiprock, NM, United States

**Keywords:** dietary assessment, dietary behavior, elementary school children, focus groups, Navajo, validity, reliability

## Abstract

**Background:**

To estimate the efficacy of interventions to improve healthy eating, valid measures are essential. Although simple dietary intake tools have been developed with other populations, few have been culturally tailored and assessed for validity and reliability among Navajo.

**Objectives:**

This study aimed to develop a simple dietary intake tool tailored to Navajo culture, derive healthy eating indices, and assess their validity and reliability in Navajo children and adults and to describe the process used to develop this tool.

**Methods:**

A picture-sort tool using typically consumed foods was developed. Elementary school children and family members provided qualitative feedback in focus groups, used to refine the tool. Next, school–aged children and adults completed assessments at baseline and follow-up. Baseline behavior measures including child self-efficacy for fruits and vegetables (F&V) were examined for internal consistency. Healthy eating indices were derived from intake frequencies from picture sorting. The convergent validity of the indices and behavior measures for children and adults were examined. The reliability of the indices at the 2 time points was derived using Bland-Altman plots.

**Results:**

The picture-sort was refined from feedback provided by the focus groups. Baseline measures from 25 children and 18 adults were obtained. In children, a modified Alternative Healthy Eating Index (AHEI) and 2 other indices from the picture-sort were correlated with self-efficacy for eating F&V and had good reliability. In adults, the modified AHEI and 3 other indices from the picture-sort had significant correlations with adult abbreviated food frequency of F&V or obesogenic dietary index and had good reliability.

**Conclusions:**

The Navajo foods picture-sort tool developed for Navajo children and adults is proven to be acceptable and feasible to implement. Indices derived from the tool has good convergent validity and repeatability, supporting use in evaluating dietary change interventions in Navajo, with the potential for broader use of the approach in other underserved populations.

## Introduction

Establishing healthy dietary choices at an early age can lead to long-term maintenance of dietary patterns. This is particularly important in communities facing significant barriers to healthy eating such as members of the Navajo Nation [1, 2, 3]. Typically, Navajo must travel far to grocery stores to purchase fresh produce because the food available at local gas stations and trading posts tends to be processed and high in salt, carbohydrate, and fat [4,5]. To address these barriers and ultimately reduce risk of obesity and diabetes, a school-based intervention to promote healthy eating and gardening among Navajo children and their families was developed [6]. Concurrently, the team created a tool to assess intervention efficacy in a subsequent study [7].

To estimate the efficacy of an intervention, accurate and robust measures of the target health behavior are essential. Methods of dietary assessment in adults have traditionally included 24-h recalls, food frequency assessment of the usual diet, and multiple days of food record [8]. Each method includes a substantial measurement error. Assessing dietary behavior in children can be particularly challenging [9] because of literacy limitations, underdeveloped sense of time, and intrusion of food preferences affecting commonly eaten foods. Several approaches have been taken to counteract these challenges, by both nutritional epidemiologists [10, 11, 12] and social and behavioral scientists [9,13,14], working to identify optimal methods to assess dietary behavior, behavioral intentions, and self-efficacy for healthy eating in children. Self-efficacy for behavior change is a well-established construct [15, 16, 17, 18, 19, 20]. The application of self-efficacy for fruits and vegetables (F&V) in children has been used in several intervention studies [13,21,22] but has not been psychometrically evaluated in a Navajo study population. Any tool adopted to estimate dietary intake or behavior should be both age and culturally appropriate and examined for validity and reliability in the population of interest [12,23].

Dietary intake has been estimated in Navajo and other American Indian children using the interviewer-administered 24-h recall [24, 25, 26], which has a high participant burden and is costly to administer. Instead of using the 24-h recall for this study, the research team sought other approaches to quantify food intake that have a lower participant burden and could be used effectively with children. Among these is the picture-sort version of the National Cancer Institute FFQ, which was initially developed for adults [11] and has been successfully used in African American children and adolescents [12,27]. Tailored pictures can include foods that are culturally appropriate and commonly consumed.

Therefore, building on long-standing collaboration between the authors and the Navajo Nation, the team used focus groups to develop a simplified dietary assessment using a picture-sort approach tailored to Navajo adults and children. This study aimed to examine the internal consistency and convergent validity of adapted child dietary self-efficacy measures, describe the process of developing the Navajo foods picture-sort frequency tool, demonstrate the feasibility of its use, and evaluate the convergent validity and reliability of its healthy eating indices.

## Methods

### Overview

**FIGURE 1:**
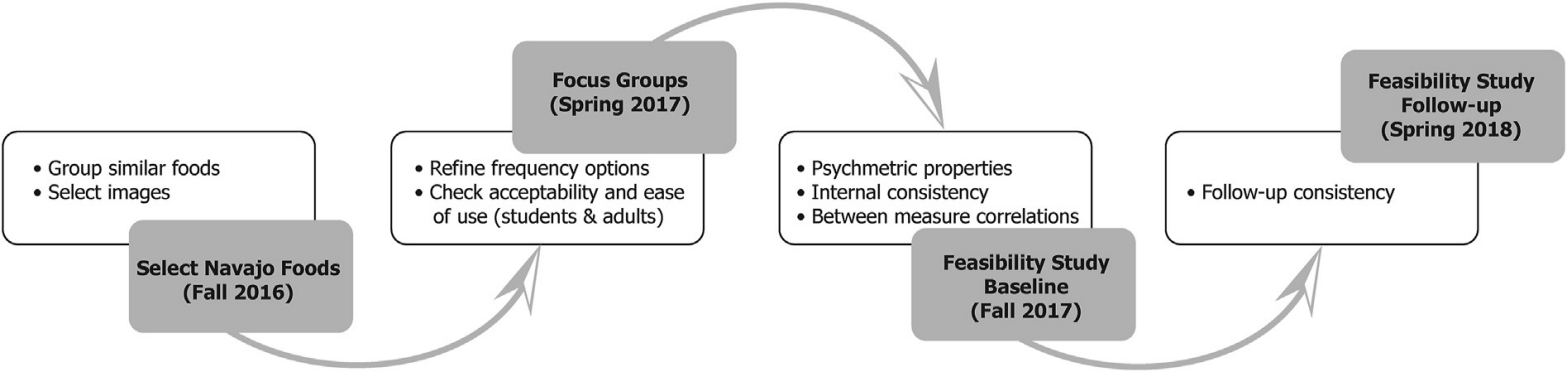
Timeline of steps in assessment development and validation.

The formative work and the Yéego! feasibility study were conducted in partnership with Dream Diné Charter School, a small school in Shiprock, NM, serving primarily Navajo children whose parents wanted instruction in Diné language and Navajo culture. Dream Diné school staff and families had expressed interest in collaborating with the research team on a school gardening and healthy eating project. The school served children from kindergarten through third grade in 2016–2017 and from kindergarten through fourth grade in 2017–2018. Shiprock, with a population of ∼9000, is located within northwest New Mexico, situated at an important road junction (US 491 and US 64) [28]. The steps in the development of the Navajo foods picture-sort and its indices used mixed methods, as outlined in Figure 1, with qualitative methods (feedback from focus groups) during the formative phase and quantitative methods at baseline and follow-up of the feasibility study. Study protocols for the focus groups and the pilot study were approved by the Fred Hutchinson Cancer Research Center Institutional Review Board and Navajo Nation Human Research Review Board. All participants provided written informed consent (and child assent).

Formative work for instrument development included interviews with teachers, focus groups with adults and children, and pretesting of assessment procedures in the first year (2016–2017). Navajo households often include extended family members, and the primary caregiver for the child may be a parent, grandparent, or other family member. They are referred to as “adult” in this article. In the second year (2017–2018), child and adult assessments in the feasibility study were conducted at the beginning (baseline) and end (follow-up) of the school year. Interviewer-administered surveys included demographic characteristics and dietary psychosocial, behavioral and intake measures. The dietary measures included self-efficacy for F&V and a “tendency to choose F&V” measure for children and an abbreviated FFQ for F&V and obesogenic dietary behavior for adults. Both children and adults completed the Navajo foods picture-sort from which dietary intake indices were derived. The psychometric properties (validity and reliability) of the dietary measures were evaluated using feasibility study baseline data. A set of final measures were recommended for use in evaluating healthy eating interventions in Navajo populations in future studies.

### Formative phase recruitment

During the formative phase of this study, flyers were distributed to children in the school (K-third grade, 2 multigrade classrooms) to solicit family participation in the focus groups. Adults and children provided consent/assent at each focus group. One focus group included kindergarten and first grade students (*n* = 7), another included second and third grade students (*n* = 8). A third focus group included adults (*n* = 13). At the end of the formative year, children and adults were again recruited from the same sampling frame as for the focus groups to complete a pretest of the dietary assessment. Consent and assent were solicited and provided as part of the survey administration.

### Navajo foods picture-sort development: children and adults

**FIGURE 2:**
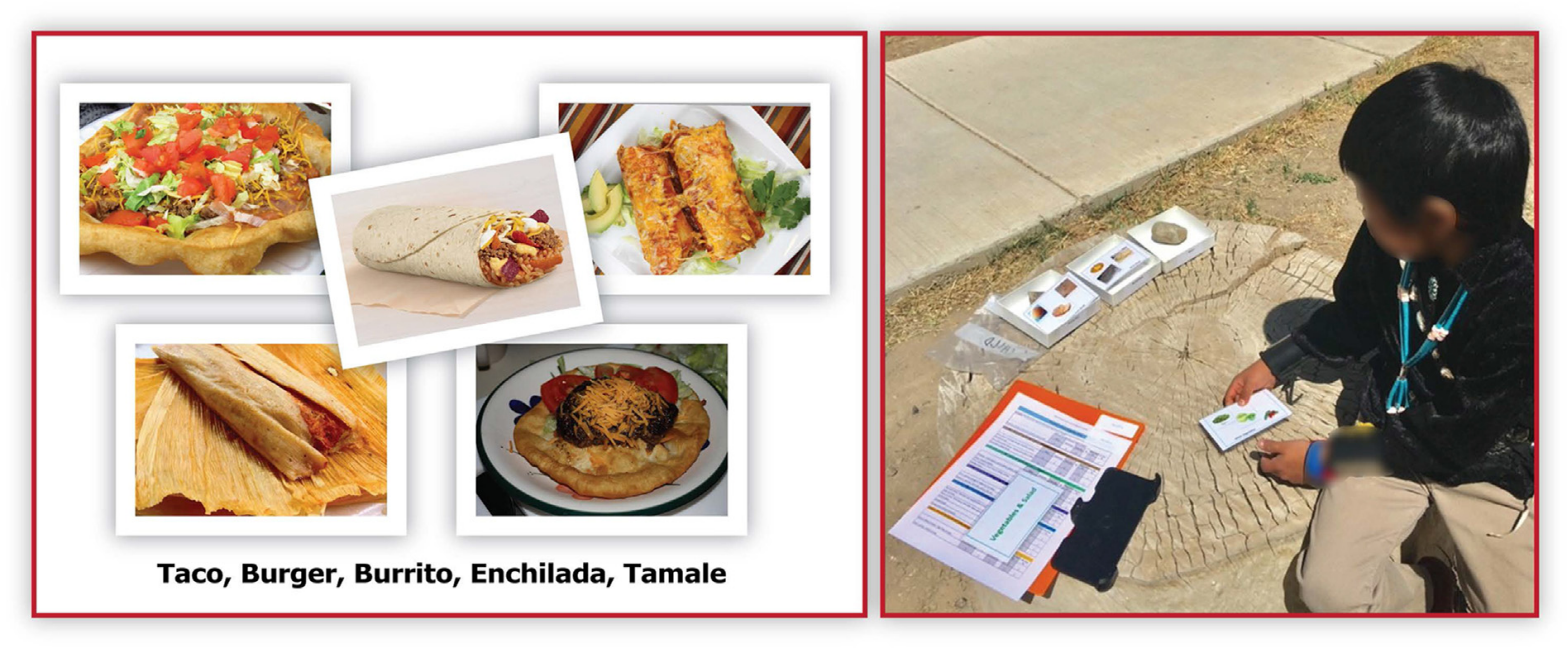
Navajo Foods Picture sort: an example of food card and use of tool in the focus group.

To begin a holistic assessment of eating patterns among Navajo elementary schoolchildren and their adults, the team modified the previously developed picture-sort methods [10, 11, 12]. First, a list was compiled of foods consumed by Navajo community members [29]. The list was grouped into categories of related foods suitable for use in a picture-sort. This Navajo foods picture-sort can be considered a simpler version of a food frequency instrument and uses photographs of commonly consumed Navajo foods [29] and other foods from local Hispanic and American cultures. An example is provided in Figure 2.

The Navajo foods picture-sort tool was introduced and discussed in the 2 child focus groups. First, the children were divided into small working groups, each with a trained facilitator. Focus groups were subjected to audio recording, and the facilitator took notes. Children were asked to sort picture cards of selected food categories in response to guiding questions, one food category at a time. Children were asked whether a food on the card was ever eaten and how frequently they usually eat it, selecting from 7 frequency options.

Feedback on the picture-sort approach and on the frequency categories was solicited during the focus groups. Some comments arose spontaneously during the focus group. Process feedback was prompted through questions such as, “What was fun about the card sort?” “Did you have trouble recognizing any of the items on the cards?” and “Were any of the foods you like to eat missing from the card sort?”

In the focus group for adults, a subset of the picture cards used with the children was evaluated. Adults were asked to sort the pictures according to how often they ate the food from 1 of the 9 response options, write down frequencies on a tracking sheet, and provide feedback on the card sort process.

After refining the picture cards and frequency options based on the qualitative feedback of the focus groups, a pretest of individual assessment was implemented for the children who provided assent. The pretest was a formal assessment, using a 2-step process applying findings from the focus groups. The first step presented picture collages of 9 food category groupings, intended to engage the children on likes and dislikes. They were asked whether they liked the foods in each group (e.g., “Do you like soups and stews?”). The main implementation of the picture-sort was the second step that included picture cards of individual food items from each category. Children were asked how often they typically ate the food item, with frequency options being never, sometimes, or every day. The sometimes category included everything in between never and at least once per day. If children indicated that a food on a card was eaten every day, they were asked if they ate the food once a day or more than once per day (Supplemental Material: Navajo foods picture-sort - Child). The pretest was used to check feasibility, completion time, and acceptability.

### Feasibility study recruitment

For the feasibility phase in the second year of the study, an information and recruitment packet was distributed through teachers to students in all grades (now kindergarten through fourth grade) in autumn 2017. All students and an adult family member were invited to participate. The adult provided written consent for themselves and their child, and the child provided assent. Participants were assessed in both autumn 2017 (baseline) and spring 2018 (follow-up). Signage and personal reminders were used to encourage completion of the assessments, which were conducted by trained interviewers on school grounds before and after the school hours.

### Measures

#### Dietary intake

The Navajo foods picture-sort tool that was implemented in the feasibility study for both children and adults covered foods that comprise 10 major food groupings of the contemporary Navajo diet [29]: breads; vegetables and salad; fruits; beverages; soups or stews; cereals; dairy and eggs; rice, pasta, etc.; meat, chicken, or fish; and desserts and snacks. At baseline, the participant’s frequency response was used to calculate servings consumed per day for every food group, ranging from 0 (never eaten) to 2 (more than once per day). A total food serving frequency was also calculated.

##### Healthy eating metrics

The reported frequencies were used to calculate modified eating scores from the applicable components of the Alternative Healthy Eating Index (AHEI) [30]. The original AHEI 2010 had 11 components and the score ranged from 0 to 110, with a mean total score of 47.6 in women and 52.4 in men [30]. Sufficient detail within the broad groupings allowed estimation of only 6 AHEI components for the modified AHEI: vegetables, fruits, whole grains, nuts and legumes, sugar-sweetened beverages and fruit juices, and red/processed meat. Consistent with the AHEI methodology, the vegetable component does not include potatoes, regardless of the preparation method. Excluded food components were *trans*-fat, long chain fats, polyunsaturated fatty acids, and sodium. Alcohol was excluded from the picture cards used by children.

In addition to a modified AHEI total score, the sum of scores from the healthy food groups (fresh and dried fruit, vegetables, whole grains, and nuts/legumes) was estimated as a healthy foods score. The subscores of fruits only, vegetables only, and F&V combined were also estimated, as is common in studies evaluating the properties of AHEI [31, 32, 33, 34]. Finally, both healthy food frequency and total food frequency were estimated. The total food frequency was obtained by summing frequencies from all questions, with the exception of alcohol in the adults, because insufficient detail was obtained. The ratio of frequency of healthy-to-total daily servings was calculated as an additional healthy eating metric. This metric is introduced to potentially correct for overreporting or underreporting by individuals, in much the same way as nutrient density can reduce bias in estimates from the FFQ [35,36].

#### Healthy eating psychosocial characteristics (children)

##### Tendency to choose F&V to eat

This measure included 6 adapted questions from a study of Hispanic youth [37,38]. Question stems used times when the child would be eating and might need to decide about F&V. Response options were modified to a 4-point Likert scale, without a neutral option. Questions about usually choosing F&V had 3 questions each. Mean scores were calculated, with higher values indicating a greater tendency to choose fruits and/or vegetables when eating. To cross-reference these measures with both the Navajo foods picture-sort indices and the self-efficacy measures, means were calculated for fruits only, vegetables only, and F&V combined.

##### Self-efficacy for F&V

This measure adapted previously published work on self-efficacy for F&V [9,39, 40, 41]. Five questions related to child’s self-efficacy to eat F&V were included in one measure. For the second measure, we added 3 questions for behavior such as bringing fruit for lunch and helping to cook vegetables. This 8-item scale was used in a study of Latino youth [22]. We modified the 4-point Likert scale response option to exclude a neutral response. In the 8-item measure, 4 items focused on fruits and 4 on vegetables (Supplemental Material: Self-efficacy questions - Child). Mean scores were calculated, with higher values indicating a greater self-efficacy. To cross-compare these measures with the Navajo foods picture-sort indices and the tendency to choose F&V to eat values, means were calculated for fruits only, vegetables only, and F&V combined (both 5-item and 8-item scores). The 5-item score was used as the reference measure for children based on its acceptance in the literature [9,13,21,22].

#### Dietary intake and behavior (adults)

##### F&V intake from abbreviated FFQ

This reference measure for adults included 7 questions originally developed for the group-randomized 5-a-Day studies [42, 43, 44] used to calculate total daily servings of F&V, excluding French fries, but including other potatoes.

##### Obesogenic dietary index

Three questions comprising the obesogenic dietary index [45] asked regarding the frequency of consuming fast food, French fries, and drinking soda (regardless of type). Frequency was converted to weekly frequency and averaged [45]. The obesogenic dietary index was used as the second reference measure for adults.

### Statistical methods

Approaches for collecting qualitative data in the formative phase include standard methods for the conduct of focus groups. Transcriptions of recordings and notes from facilitators were reviewed. Feedback was categorized into those concerning the pictures or choice of foods shown in the Navajo foods picture-sort and those concerning the selection of frequency options. Children were observed during the practice card-sorting, and difficulties were noted.

In the feasibility study phase, feasibility and acceptability were measured by the proportion of respondents completing the follow-up assessments. Response rates of two-thirds or greater were considered as success. Demographic characteristics for students and adults were described for those with baseline data and for subsets with both baseline and follow-up data. Means and SDs and percentages were estimated for continuous and categorical variables, respectively. Acceptability, usability, and ability to retain respondents to complete the instrument a second time were examined descriptively.

#### Refining child healthy eating psychosocial measures

Self-efficacy for F&V and tendency to choose F&V measures were evaluated for internal consistency among the Navajo children, using baseline data from the survey. A list of possible indices from the Navajo foods picture-sort were prespecified (see the Healthy eating metrics section). The ranges of all measures of healthy eating behavior and psychosocial variables were estimated and checked for variability. Because the theoretical ranges differed between the Navajo foods picture-sort indices and the psychosocial measures, variables were rescaled to be between 0 and 1 by subtracting the lowest possible value and dividing by the possible range, to allow for the comparisons of variability using the coefficient of variation. For example, the self-efficacy scale had a possible range of 1 to 4, so values were rescaled by subtracting 1 and dividing by 3. The reliability (internal consistency) of each psychosocial scale was examined using Cronbach *α*. A single item was removed from a scale if the resulting internal consistency improved by >30%.

#### Validity and test-retest reliability of healthy eating measures

Correlations between the revised child healthy eating psychosocial scores and Navajo foods picture-sort intake indices were calculated to assess convergent validity. Healthy eating indices from the Navajo foods picture-sort that had nonstatistically significant correlations (*P* > 0.1) with the self-efficacy for eating F&V score (reference measure for children) were not retained for further evaluation. Similar investigation of convergent validity used the established F&V intake measures for adults from the abbreviated FFQ and the obesogenic dietary index [45]. Correlations between these and the healthy eating indices from the Navajo foods picture sort were calculated. The obesogenic dietary index collected in adults was expected to be negatively correlated with F&V measures and with the ratio of healthy foods daily servings to total foods daily servings estimated from the Navajo foods picture-sort frequencies. Again, only adult Navajo foods picture-sort indices that were significantly correlated with the adult reference measures were retained.

Using values from the beginning and end of the school year (baseline and follow-up), differences were plotted against means at the 2 time points on a Bland-Altman plot [46] for each of the retained child and adult measures. The limits of agreement were calculated using the variance of differences [47], namely mean difference ± 1.96 × SD (difference). The proportion of differences within the limits of agreement was calculated from the plot. The reliability coefficient [48] was calculated using the within-person variance.

## Results

### Qualitative results from the focus groups

The child picture-sort originally had 7 frequency options, but because the children had difficulty selecting from so many options during the first focus group, it was simplified to the frequency options “never,” “once per week,” and “once per day” as the focus group continued. Then, the once per day option was revised to “every day” to make it simpler, and a further step distinguishing “once a day” from “more than once a day” was added. Two pictures, one of a Navajo food and one of a Mexican food, were not recognized as the food they represented. Feedback included, “I didn’t understand menudo soup, it doesn’t look like that.” We replaced these pictures in the final version. The “once per week” category was confusing to some children without the context that this was supposed to represent less frequent than once per day.

Findings from the focus group with adults indicated too many overlapping frequency options and too many pictures per category. Therefore, similar foods were combined into 1 picture card, and frequency options for adults were reduced to 5: “never,” “more than once per day,” “every day,” “weekly,” and “sometimes but not weekly.” Apart from the number of frequency options, the final adult and child versions of the Navajo foods picture-sort were identical.

The pretest of the surveys was completed by 21 children and 16 adults. There were no missing or incomplete responses to questions, including the responses for the revised Navajo foods picture-sort completed by the children. It took ∼20 min for the child to complete the picture-sort questions. In addition, the respondents reported no difficulty in understanding the questions asked. Based on these observations, we determined that the assessments were feasible and acceptable to our respondents.

### Feasibility study

The proportion of child and adult baseline respondents who completed the assessments at follow-up was 72% and 67%, respectively. Baseline demographic characteristics and descriptive statistics of the dietary measures for the 25 children and 18 adults who completed baseline assessment are summarized in Table 1. Approximately 90% of both child and adult responders spoke some Navajo, either exclusively or with intermittent English spoken in the home. From the Navajo foods picture-sort frequencies, it seems that both children and adults reported consuming just over 3 servings/d of F&V (excluding juice). In adults, this estimate was similar to that obtained from the NCI 7 summary questions (3.0) that form the abbreviated FFQ measure of F&V intake [8]. Responses to the modified AHEI score ranged from 1.5 to 35.

**TABLE 1:** Baseline demographic and dietary characteristics of children and adults∗

Characteristics	Children (*n* = 25)	Adults (*n* = 18)
Females	32	78
American Indian or Alaska Native	100	89
Only Navajo spoken at home	21	—
Both Navajo and English spoken at home	71	—
Reads and speaks Navajo	—	89
Has some college or college graduation	—	83
Married	—	35
Relationship to child—mother	—	61
Age (y)	7.2 (1.4)	35 (8.9)
Picture-sort frequency tool		
Fruits only (servings/d)	1.5 (0.9)	1.8 (1.1)
Vegetables only (servings/d)	1.6 (1.1)	1.7 (0.95)
F&V, excluding fruit juice (servings/d)	3.2 (1.6)	3.5 (1.8)
Ratio of healthy-to-total daily servings	0.25 (0.06)	0.39 (0.13)
F&V Score	7.1 (3.5)	7.8 (4.2)
Healthy foods score	18.1 (6.2)	16.3 (6.4)
Modified AHEI total score	19.0 (6.2)	20.4 (8.4)
Adult survey only		
FFQ fruits only (servings/d)	—	2.0 (1.5)
FFQ vegetables only (servings/d)	—	1.0 (1.3)
FFQ F&V (servings/d)	—	3.0 (1.8)
Obesogenic dietary index	—	2.5 (1.7)

AHEI, Alternative Healthy Eating Index.

∗ Values are presented as percentage or mean (SD).

### Internal consistency of behavioral measures

As tabulated in Table 2, overall baseline F&V psychosocial measures had ranges of values between 1.5 and 4.0 and mean values above the midpoint. Rescaling both frequency measures and psychosocial measures to between 0 and 1 revealed that self-efficacy measures had slightly lower coefficients of variation than either servings per day from the Navajo foods picture-sort method or tendency to choose to eat scores (data not shown). A Cronbach *α* score of ≥0.6 was observed for all tendency to choose scores, but only for the combined F&V scores for self-efficacy (Table 2). Self-efficacy for fruits score had a Cronbach *α* of 0.31 that improved to 0.67 by deleting the item “… add fruit to your cereal for breakfast.” Similarly, the 4-item vegetable self-efficacy score improved to 0.50 with the exclusion of the item “… eat a serving of vegetables for dinner.” The 5-item self-efficacy for eating F&V and the 8-item expanded self-efficacy for F&V each had a good internal consistency, and neither improved substantially by deleting 1 item. These 2 measures, together with the 2-item self-efficacy for eating fruits score and the 3-item self-efficacy for eating/bringing/cooking vegetable score, were used in the subsequent psychometric evaluation.

**TABLE 2:** Distribution and internal consistency of child healthy eating psychosocial measures at baseline (*n* = 25)

Measures (No. of items)	Mean ± SD	Range	Cronbach *α*	Cronbach *α* after excluding 1 item	The item excluded
Tendency to choose to eat					
Fruits only (3)	2.7 ± 0.9	1.3–4.0	0.60	0.62	Eat fruit during snack time
Vegetables only (3)	2.7 ± 0.9	1.0–4.0	0.73	0.76	Eat vegetable at lunch
F&V (6)	2.7 ± 0.7	1.5–3.7	0.66	0.69	Eat vegetable at lunch
Self-efficacy for F&V					
Fruits, eating (3)	3.0 ± 0.6	1.7–4.0	0.31	0.67	Add fruit to my cereal for breakfast
Fruits, including bringing to school (4)	3.0 ± 0.5	1.5–4.0	0.40	0.49	Add fruit to my cereal for breakfast
Vegetables, eating (2)	3.2 ± 0.6	1.5–4.0	0.03	NA	
Vegetables, including bringing and cooking (4)	3.1 ± 0.5	1.8–4.0	0.30	0.50	Eat a serving of vegetables for dinner
F&V, eating (5)	3.1 ± 0.5	1.6–3.8	0.60	0.65	Add fruit to my cereal for breakfast
F&V, including bringing and cooking (8)	3.1 ± 0.5	1.9–3.8	0.64	0.66	Bring a lunch to school with a vegetable

F&V, fruits and vegetables; SD, standard deviation.

### Convergent validity

Correlations of the Navajo foods picture-sort metrics with self-efficacy scores were consistently higher than those with tendency to choose to eat scores (Table 3). For example, the correlation of the Navajo foods picture-sort F&V score with the tendency to choose to eat F&V score was 0.18 but was 0.31 with the self-efficacy 5-item measure for F&V. Moreover, the correlations between the tendency to choose F&V to eat scores and the self-efficacy scores were low. Based on these low correlations, the tendency to choose measure was not retained for the subsequent psychometric evaluation. Healthy foods score, modified AHEI total score, and ratio of healthy-to-total daily servings correlated at ≥0.38 with 1 or more of the self-efficacy for F&V scores. Among adults, daily F&V servings from the abbreviated FFQ positively correlated with the Navajo foods picture-sort fruit frequency score (0.73), the F&V frequency score (0.66), the healthy food score (0.57), and the modified AHEI total score (0.47). The obesogenic dietary index correlated negatively with the Navajo foods picture-sort derived ratio of healthy-to-total daily servings and with F&V frequency score, including with its subcomponents (Table 3). The results for both children and adults found that the indices from the Navajo foods picture-sort that were deemed to have acceptable psychometric properties were modified AHEI total score, healthy food score, and ratio of healthy-to-total daily servings.

**TABLE 3:** Pearson correlation matrix measures of fruits and vegetables and other dietary behaviors

	**Picture-sort** measures						Tendency to choose to eat		
	Ratio of healthy-to-total daily servings	Fruit score	Vegetable score	Fruit and vegetable score	Healthy foods score^1^	Modified AHEI total score	Fruits only	Vegetables only	Fruits and vegetables
**Children (*n* = 25)**									
**Tendency to choose to eat**									
Fruits only	0.05	0.22	0.12	0.21	0.30	0.32	1	0.24	0.79
Vegetables only	−0.07	−0.19	0.30	0.07	0.04	0.02		1	0.78
Fruits and vegetables	−0.01	0.02	0.27	0.18	0.22	0.22			1
**Self-efficacy for fruits and vegetables**									
Fruits (eat: lunch, dessert)	0.39^2^	0.46^2^	0.14	0.39^3^	0.45^2^	0.39^2^	0.06	−0.02	0.03
Vegetables (eat served lunch, bring for lunch, help cook)	0.12	−0.02	0.47^2^	0.28	0.29	0.28	−0.16	0.08	−0.05
Eat fruits and vegetables (5 items including add fruit to my cereal for breakfast)	0.38^3^	0.30	0.18	0.31	0.42^2^	0.41^2^	0.14	0.26	0.25
Eat/bring/cook fruits and vegetables (all 8 items)	0.33	0.12	0.34^3^	0.29	0.36^3^	0.34^3^	−0.02	0.14	0.07
**Adults (*n* = 18)**									
**Abbreviated FFQ**									
Fruits only	0.19	0.62^4^	0.02	0.42^3^	0.28	0.38			
Vegetables only	−0.14	0.27	0.46^3^	0.39^3^	0.45^5^	0.19			
Fruits and vegetables	0.06	0.73^6^	0.36	0.66^2^	0.57^4^	0.47^2^			
Obesogenic dietary index	−0.59^4^	−0.52^2^	−0.48^2^	−0.57^4^	−0.23	−0.42^3^			

AHEI, Alternative Healthy Eating Index.

1 Fruits (fresh and dried), vegetables, whole grains, beans, and nuts.

2 *P* < 0.05.

3 *P* < 0.1.

4 *P* ≤ 0.01.

5 *P* < 0.001.

### Reliability

The means of measures at baseline and follow-up are summarized in Table 4 for both children and adults. Moreover, the test-retest reliability results of the indices from the Navajo foods picture-sort are tabulated in Table 4. These were derived from Bland-Altman plots (not shown) and included the repeatability coefficient, the limits of agreement, and the percentage of test-retest differences falling between the limits of agreement. The percentage, which was expected to be 95%, was 94% for each of the child healthy eating indices and between 92% and 100% for the adult healthy eating indices.

**TABLE 4:** Child and adult healthy eating measures for those completing both baseline and follow-up∗

	Baseline	Follow-up	Repeatability coefficient	Limits of agreement	%Difference between baseline and follow-up values falling within limits of agreement
Children (*n* = 18)					
Self-efficacy to eat F&V (5 items)	3.3 (0.3)	3.3 (0.5)	—	—	—
Self-efficacy to bring, cook, etc, F&V (8 items)	3.2 (0.5)	3.2 (0.4)	—	—	—
Healthy foods^1^ score	19.1 (6.0)	17.7 (6.0)	15.3	−16.8, 14.2	94
Modified AHEI total score	20.0 (6.3)	18.8 (7.6)	19.1	−20.7, 18.4	94
Ratio of healthy-to-total daily servings	0.3 (0.1)	0.2 (0.1)	0.23	−0.25, 0.21	94
Adults (*n* = 12)					
FFQ F&V servings/d	2.9 (1.7)	2.3 (1.7)	—	—	—
Obesogenic dietary index	2.8 (2.0)	3.2 (1.8)	—	—	—
F&V score	7.1 (4.5)	7.2 (4.3)	9.0	−9.3, 9.5	92
Healthy foods^1^ score	15.8 (5.9)	16.1 (7.1)	13.0	−13.4, 13.8	100
Modified AHEI total score	20.6 (7.9)	18.8 (6.6)	15.6	−17.7, 14.1	92
Ratio of healthy-to-total daily servings	0.4 (0.1)	0.4 (0.1)	0.25	−0.27, 0.27	92

AHEI, Alternative Healthy Eating Index; F&V, fruits and vegetables.

∗ Values are presented as percentage or mean (SD).

1 Fruits (fresh and dried), vegetables (but not salad), whole grains, beans, and nuts.

## Discussion

We successfully developed a Navajo foods picture-sort tool for assessing healthy eating in the Navajo Nation and used the tool in a feasibility study. The tool was feasible and acceptable to both elementary school–aged children and their adult family members, as evidenced by the proportion who completed the follow-up assessments in the feasibility study. The baseline assessments were successfully used to evaluate the internal consistency and convergent validity among Navajo children, resulting in a refined healthy eating psychosocial measure, the self-efficacy for eating F&V.

The main lessons learned from the process of developing the Navajo foods picture-sort may have applicability to other indigenous or underserved communities. Earlier work with key influentials in the Navajo communities of Shiprock and Tsaile highlighted many of the typical foods eaten by these communities. Identifying the most relevant foods in these communities in interviews with the teachers and staff before the focus groups established relationships with the team, thereby facilitating the collaboration during the feasibility study. The steps taken to check whether the pictures of those foods were recognized by the children and the adults were essential to include a broad span of frequently eaten foods. Similarly checking that the wording of the frequency options was salient to both the children and the adults was helpful in finalizing the categories. Engaging the children in talk about foods they like enhanced rapport with the interviewer and encouraged participation in the frequency assessment using the Navajo foods picture-sort tool. The modifications to the Navajo foods picture-sort tool during the development process contributed to the successful implementation of the tool and its metrics in the feasibility study.

The Navajo foods picture-sort frequency tool showed strong concordance with other established measures of dietary intake and behavior in both children and adults in our study, with correlations >0.4 indicating convergent validity. These findings align with those of the previous studies. For example, 2 studies in African American children showed strong correlations between Navajo foods picture-sort derived estimates of macronutrients and estimates from 24-h recalls (∼0.6 for carbohydrates) [12] and estimates from food preferences (between 0.3 and 0.4) [27]. Another small validation study of a 1-d frequency consumption form (Yesterday’s Food Choices) among fifth-grade and seventh-grade American Indian children found a Spearman rank correlation with a single 24-h recall to be 0.29 for F&V [25]. Other shortcut measures have been applied in the studies of elementary school–aged children [21,49, 50, 51] and, particularly, American Indian children [52]. The scoring system used in this Yéego! study was based on the weights (scores) from the adult AHEI-2010 method described by Chiuve et al. [30]. Others have developed different weights for scoring healthy eating components for children and youth. For example, Feskanich et al. [53] used 3 or more servings per day of fruits for the maximum score of 10 as one component of their Youth Healthy Eating Index, compared with the 4 or more servings required for the maximum score as part of the AHEI. Similarly, 3 or more servings of vegetables were scored 10 in the Youth Healthy Eating Index compared with 5 or more servings of vegetables scored as 10 in the AHEI. These new scoring weights proposed had some differences from the USDA Dietary Guidelines for 2000 [54], an accepted standard at the time of publication of that article. More recently, a global review of diet quality indices in children and adolescents [55] included findings from 22 studies using adaptions of the Healthy Eating Index that evaluated their indices for validity and/or reliability. Several recommendations emerged from the review, including to select a dietary assessment method that can be implemented easily in practice; to select a diet quality index whose scoring reflects a nutritional reference standard relevant to the population of interest; and to evaluate the validity and /or reliability of the index in the study population. These recommendations were followed by this study.

A recent systematic review of obesity prevention among indigenous children worldwide [56] included 3 studies in Navajo elementary schools [57, 58, 59]; however, only 1 used estimates of dietary intake and behavior to evaluate their intervention program. The intervention was associated with gains in food self-efficacy [57] and a lower percentage of energy from fat intake [60]. These studies in Navajo children mainly used the 24-h recall method for dietary assessment. In the Pathways feasibility study, children were given instructions on portion sizes and how to keep a semiquantitative food record [24,25] to prepare them for completing a 24-h recall. The 24-h recall method is best administered by a trained interviewer and is designed to collect sufficient detail to estimate macronutrient and micronutrient content. Therefore, this method is more burdensome and expensive to implement, and studies often choose to obtain it in only a subset of participants [25,57,60].

By contrast, food frequency tools with pictures are easy to use, can capture the approximate frequency of foods consumed regularly and can evaluate the contribution of a food category in the context of overall dietary pattern. Summary indices, such as the ratio of healthy-to-total daily servings or the modified AHEI total score, can be derived from these tools and reflect foods that are consumed in patterns, consistent with local norms. In particular, these 2 indices may have the advantage of being less susceptible to bias from overreporting or underreporting tendencies because they correct for overall reporting (the ratio) or balance out the nonhealthy foods (the AHEI total score). They are also in line with the holistic approach taken currently by the recent Dietary Guidelines for Americans [61], which focuses on food and beverage combinations, rather than food categories.

Furthermore, the Navajo foods picture-sort frequency tool can be completed by children with minimum supervision and in a short period, reducing burden and cost. Compared to 24-h recalls, the Navajo foods picture-sort tool was designed particularly for cultural inclusivity and is more affordable for collecting, coding, and analyzing dietary intake measures in studies with larger sample sizes and in younger children.

In addition to the ability of the Navajo foods picture-sort tool to directly estimate food intake and dietary patterns in children, the summary indices can be used with other dietary psychosocial or behavior measures to support inferences from a single measure. Our self-efficacy with F&V measure had good internal consistency (comparable with self-efficacy to eat a healthy diet reported in the Pathways study [57]) and concordance with F&V consumption measures, including from the Navajo foods picture-sort. In this study, the baseline mean self-efficacy scores were consistent with the mean scores of a 14-item F&V self-efficacy measure collected from similar-aged, urban Latino children, also enrolled in a gardening, nutrition, and cooking intervention [22]. Regarding the tendency to choose scale, this study found higher mean scores than those baseline scores reported in a sample of rural, Hispanic children [38]. In contrast to the self-efficacy for F&V scores, the tendency to choose scale had a high internal consistency, but its low correlation with other F&V measures suggests that the wording of “I usually choose to eat…” may have been interpreted differently by Navajo children (in contrast to Hispanic children). Although, in Hispanic children, this scale was considered to be a measure of self-efficacy, in this study, it does not adequately capture self-efficacy and may be measuring a different construct than the other F&V measures.

The study has several limitations. The continuity of students in the charter school from the first (formative) year was unexpectedly low (6 students), limiting our ability to estimate test-retest reliability of our psychosocial measures. The total number of children completing baseline in the second year was also small (25 children in the Fall, and 22 children—including 4 who did not respond at baseline—in the following spring). Several elements of the AHEI [30] could not be estimated from the picture-sort tool, which may have reduced the precision of the modified AHEI total score, the ratio of healthy-to-total foods daily servings, and the healthy foods score. As with many other behavioral change evaluations, all outcome measures are based on self-report and are, thus, susceptible to bias. The generalizability of the study may be limited by the fact that this was a small charter school. Experience with larger elementary schools on the Navajo Nation may be different.

Parallel development of intervention and instrumentation, a process used in other studies of indigenous peoples [26,58], was a study strength. Additional strengths were the use of the Navajo foods picture-sort with its indices concordance with other validated measures of dietary behavior in children and adults [8,42, 43, 44, 45], its inclusion of special foods unique to the Navajo culture, and its ease of use among both Navajo and English speakers. The established relationships with the Navajo communities greatly enhanced this study.

In summary, we developed and tested a culturally appropriate food picture-sort frequency tool that is feasible and acceptable to both Navajo children and adults. This tool and its indices have the potential to measure the change for school-based intervention studies among the Navajo Nation because of its cultural appropriateness, ease of administration and low burden, and the convergent validity and reliability of its indices. Although the tool was developed for studies with Diné people, the approach to tailoring diet quality indices to culture and age groups can be applied in other populations. More research is needed to see whether the tool and its indices are sensitive enough to assess the behavioral changes in groups attributable to these types of interventions. Next steps include testing these measures and implementing and evaluating the intervention in a larger study on the Navajo Nation.

## Acknowledgments

We thank the staff at Office of Dream Diné Charter School (Shiprock) and the New Mexico State University and AmeriCorps staff members who assisted in the data collection. We extend our thanks to the Navajo Nation Human Research Review Board and Shiprock Chapter House for reviewing the study.

## Author contributions

SAAB, IJO, MCB, KAL: designed the research; KR, SKB, DD, IJO: conducted the research; ERS, SAAB: performed the statistical analysis; SAAB, ERS: wrote the paper; SB: had primary responsibility for the final content; and all authors: read and approved the final manuscript.

## Data availability

The data described in the manuscript, code book, and analytic code will be made available on request pending application to and approval by the Navajo Nation Human Research Review Board. The data belong to the Navajo Nation, which regulates access to data regarding Navajo people collected on the Navajo Nation. The Navajo Nation values results of collaborative research being available broadly through publication in the scientific literature.

## Funding

This project is with the Partnership for the Advancement of Cancer Research, supported in part by NCI grants U54 CA132383 (NMSU) and U54 CA132381 (Fred Hutch).

## Author disclosures

SAAB, ERS, KR, SKB, DD, IO, MCB, and KL have no conflicts of interest to report.

The funding sources had no role in the study design; collection, analysis and interpretation of data; or writing of the report and had no restrictions regarding publication.

## Appendix A Supplementary data

Multimedia component1 Multimedia component1
