# Supplementary material for: Development of an Assessment Tool to Measure Healthy Eating in Navajo Children and Their Families
Source: Curr Dev Nutr. 2023 Apr 1;7(5):100074. doi: 10.1016/j.cdnut.2023.100074 (PMC10192404; doi:10.1016/j.cdnut.2023.100074)
Supplement: Multimedia component1 [file mmc1.pdf]

Development of an assessment tool to measure healthy eating in Navajo children and their families

Beresford, S.A.A.

Supplementary material: Food and drink items listed Parent Cardsort

Card Sort - PARENT_Collection Sheet

Score

EXAMPLE: This person ate macaroni and cheese about once a month.

HOW OFTEN DID YOU EAT THESE FOODS?

	Never	More than Once per Day	Every Day	Weekly	Sometimes But Not Weekly
Macaroni cheese or fettuccine alfredo or pasta salad	☐ 0	☑ 2	☐ 1	☐ 1.57	☐ 0.79

BREADS - HOW OFTEN DID YOU EAT THESE FOODS?	Never	More than Once per Day	Every Day	Weekly	Sometimes But Not Weekly
White Bread - fry bread, Navajo white flour tortilla, blue corn bread	☐	☐	☐	☐	☐
Wheat/Whole Grain Bread - Pueblo bread, Navajo whole wheat tortilla, wheat bread	☐	☐	☐	☐	☐

VEGETABLES AND SALAD - HOW OFTEN DID YOU EAT THESE FOODS?	Never	More than Once per Day	Every Day	Weekly	Sometimes But Not Weekly
Potatoes - Fried potato, mashed potato, potato salad	☐	☐	☐	☐	☐
Salad - Salad & Coleslaw	☐	☐	☐	☐	☐
Other Vegetables - Green beans, chilies, spinach, cabbage greens	☐	☐	☐	☐	☐

FRUITS - HOW OFTEN DID YOU EAT THESE FOODS?	Never	More than Once per Day	Every Day	Weekly	Sometimes But Not Weekly
Fruit - banana, orange or tangerine, fruit salad	☐	☐	☐	☐	☐
Raisins or Trail Mix - Dry fruits, raisins, trail mix	☐	☐	☐	☐	☐

SOUPS OR STEWS - HOW OFTEN DID YOU EAT THESE FOODS?	Never	More than Once per Day	Every Day	Weekly	Sometimes But Not Weekly
Red Meat Stew - Menudo stew, posole/hominy stew with red meat	☐	☐	☐	☐	☐
Beans or Chile - Pinto beans with chili, beans (plain), refried beans	☐	☐	☐	☐	☐
Navajo burger	☐	☐	☐	☐	☐
Navajo roast mutton sandwich	☐	☐	☐	☐	☐

CEREALS - HOW OFTEN DID YOU EAT THESE FOODS?	Never	More than Once per Day	Every Day	Weekly	Sometimes But Not Weekly
Bran or Wheat Cereals - High fiber cereals	☐	☐	☐	☐	☐
Other Cereals - Sweet cereals	☐	☐	☐	☐	☐

DAIRY AND EGGS - HOW OFTEN DID YOU EAT THESE FOODS?	Never	More than Once per Day	Every Day	Weekly	Sometimes But Not Weekly
Milk (1%) or skimmed	☐	☐	☐	☐	☐
Other Milk - Milkshake, whole milk, 2% milk	☐	☐	☐	☐	☐
Eggs, cheese yogurt	☐	☐	☐	☐	☐

RICE, PASTA, ETC. - HOW OFTEN DID YOU EAT THESE FOODS?	Never	More than Once per Day	Every Day	Weekly	Sometimes But Not Weekly
Taco burger, burrito, enchilada, tamale - Navajo taco, Navajo burger; burrito	☐	☐	☐	☐	☐
Fried rice, rice	☐	☐	☐	☐	☐

MEAT CHICKEN OR FISH - HOW OFTEN DID YOU EAT THESE FOODS?	Never	More than Once per Day	Every Day	Weekly	Sometimes But Not Weekly
Processed meat, including Spam - Bologna, salami, lunch meat or processed meat	☐	☐	☐	☐	☐
Red Meat - Meatloaf, ground beef, pork chops	☐	☐	☐	☐	☐
Chicken - Any chicken fried, any chicken baked, roasted etc.	☐	☐	☐	☐	☐
Fish - Tuna or tuna salad, any fish, shrimp	☐	☐	☐	☐	☐

DESSERTS AND SNACKS - HOW OFTEN DID YOU EAT THESE FOODS?	Never	More than Once per Day	Every Day	Weekly	Sometimes But Not Weekly
Dessert - Ice cream, apple pie, cake	☐	☐	☐	☐	☐
Muffin or fruit bread - Muffin, fruit cake, banana bread	☐	☐	☐	☐	☐
Cookie - cookies, rice krispie treat	☐	☐	☐	☐	☐
Nuts - Nuts, peanut butter	☐	☐	☐	☐	☐

BEVERAGES - HOW OFTEN DID YOU EAT THESE FOODS?	Never	More than Once per Day	Every Day	Weekly	Sometimes But Not Weekly
Sweetened Drinks - Regular soft drinks, fruit punch	☐	☐	☐	☐	☐
Fruit Juice - 100% orange juice, 100% apple juice	☐	☐	☐	☐	☐
Alcohol, Bear, Wine, Spirits	☐	☐	☐	☐	☐

Supplementary material: Navajo foods picture-sort - Child

Hello! Thank you for participating! In this survey, you will be answering questions about foods you eat. You are going to read each question and all of the possible answers very carefully, and then you will choose ONE answer. You will check off the answers on your own, so make sure to mark what you think, not what anybody else thinks. There are no right or wrong answers. PLEASE MARK ONLY ONE ANSWER.

1. How often do you eat **White Bread** such as: blue corn bread, fry bread, Navajo white flour tortilla?

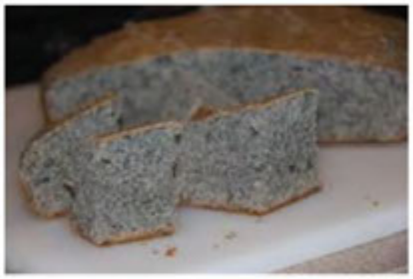
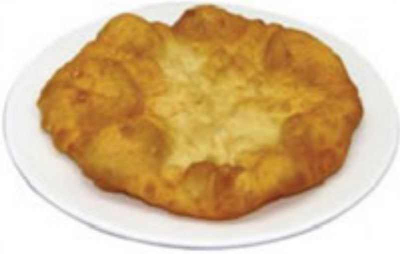
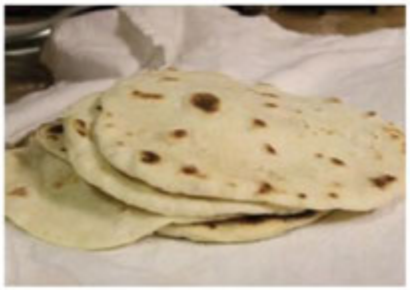

☐ **Never** ☐ **Sometimes** ☐ **Every day** → If every day, more than 1 per day?
☐ **Yes** ☐ **No**

2. How often do you eat **Wheat/Whole Grain Bread** such as: Pueblo bread, Navajo whole wheat tortilla, wheat bread?

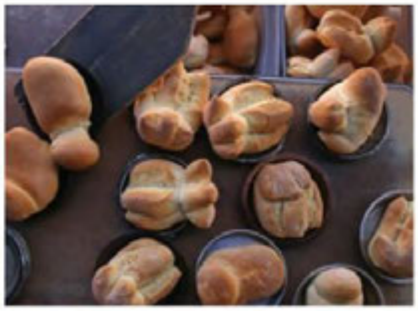
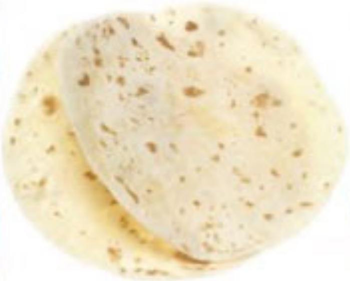
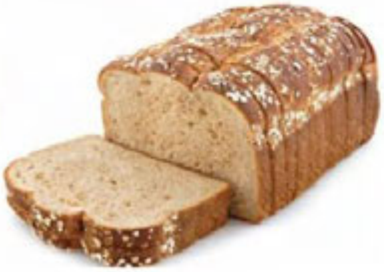

☐ **Never** ☐ **Sometimes** ☐ **Every day** → If every day, more than 1 per day?
☐ **Yes** ☐ **No**

3. How often do you eat **Potatoes** such as: mashed potato, fried potato, potato salad?

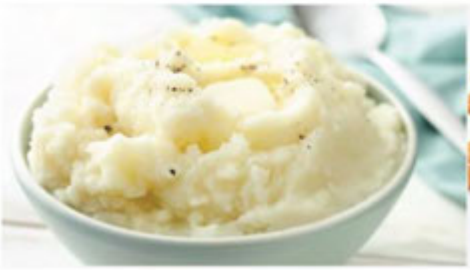
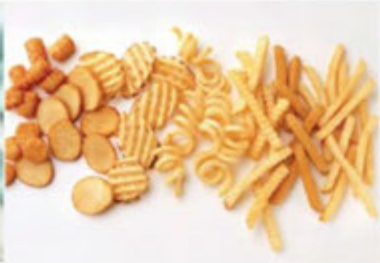
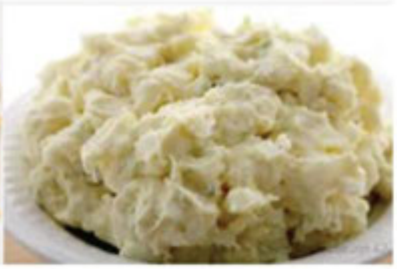

☐ **Never** ☐ **Sometimes** ☐ **Every day** → If every day, more than 1 per day?
☐ **Yes** ☐ **No**

4. How often do you eat **Salad** such as: green salad & coleslaw

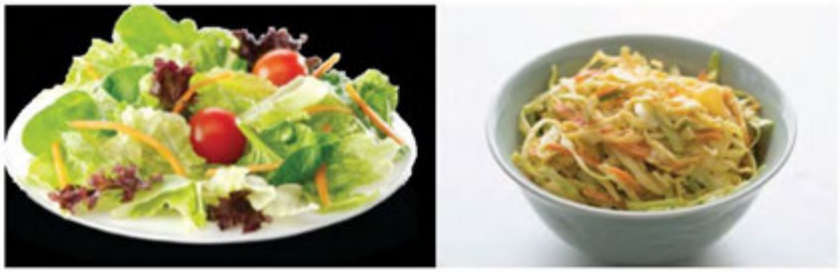

☐ **Never** ☐ **Sometimes** ☐ **Every day** → If every day, more than 1 per day?
☐ **Yes** ☐ **No**

5. How often do you eat **Other Vegetables** such as: spinach, cabbage greens, green beans, chilies?

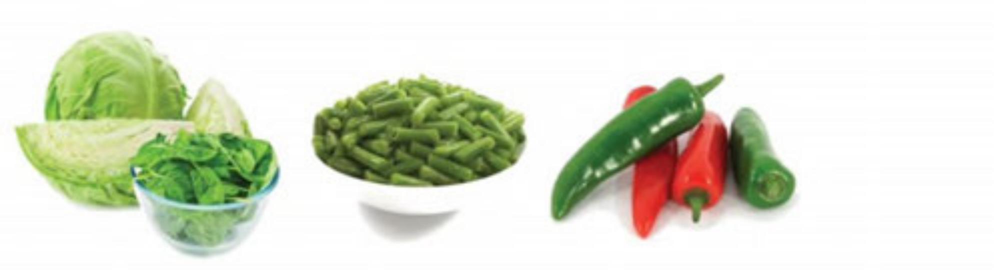

☐ **Never** ☐ **Sometimes** ☐ **Every day** → If every day, more than 1 per day?
☐ **Yes** ☐ **No**

6. How often do you eat **Fruit** such as: Banana, fruit salad, orange or tangerine

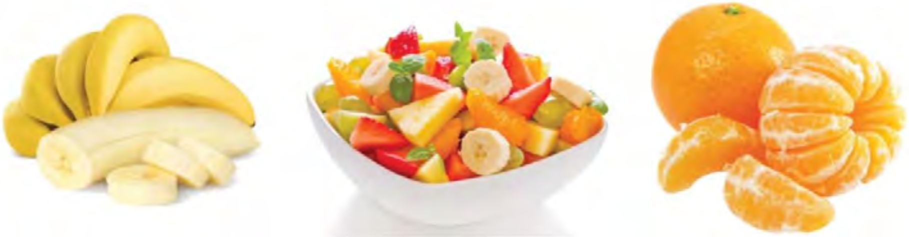

☐ **Never** ☐ **Sometimes** ☐ **Every day** → If every day, more than 1 per day?
☐ **Yes** ☐ **No**

7. How often do you eat **Raisins or Trail Mix** such as: Dry fruits including raisins, trail mix?

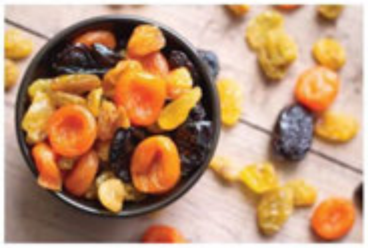

☐ **Never** ☐ **Sometimes** ☐ **Every day** → If every day, more than 1 per day?
☐ **Yes** ☐ **No**

8. How often do you eat **Red Meat Stews** such as: Menudo stew, posole/hominy stew with red meat?

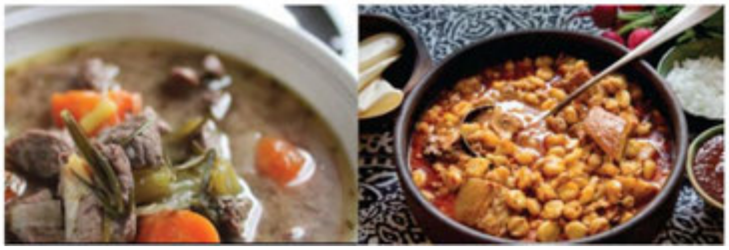

☐ **Never** ☐ **Sometimes** ☐ **Every day** → If every day, more than 1 per day?
☐ **Yes** ☐ **No**

9. How often do you eat **Beans or Chili** such as: pinto beans with chili, refried beans, beans (plan)

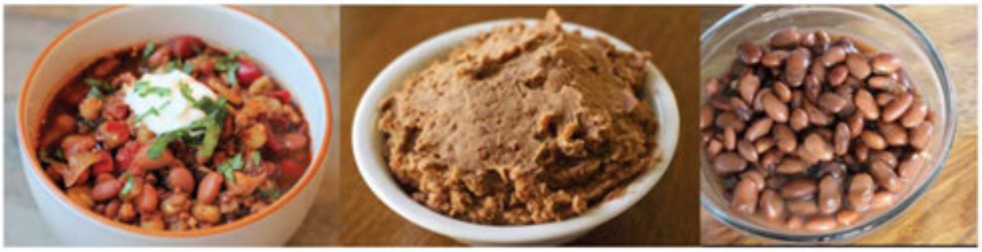

☐ **Never** ☐ **Sometimes** ☐ **Every day** → If every day, more than 1 per day?
☐ **Yes** ☐ **No**

10. How often do you eat **Bran or Wheat Cereals** such as: high fiber cereals?

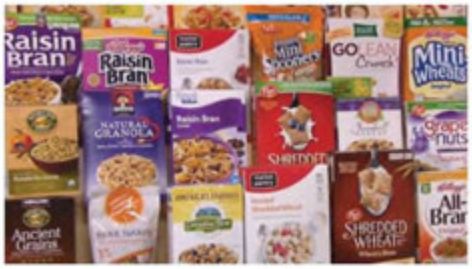

☐ **Never** ☐ **Sometimes** ☐ **Every day** → If every day, more than 1 per day?
☐ **Yes** ☐ **No**

11. How often do you eat **Other Cereals** such as: sweet cereals?

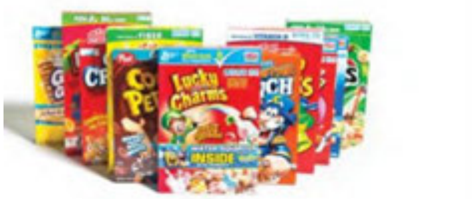

☐ **Never** ☐ **Sometimes** ☐ **Every day** → If every day, more than 1 per day?
☐ **Yes** ☐ **No**

12. How often do you think **Skim Milk** such as: fat free or skim milk?

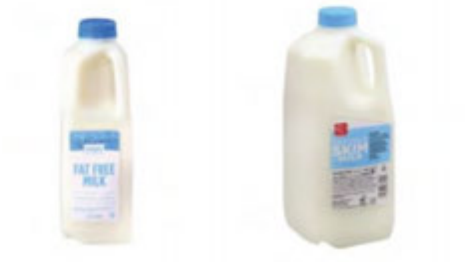

☐ **Never** ☐ **Sometimes** ☐ **Every day** → If every day, more than 1 per day?
☐ **Yes** ☐ **No**

13. How often do you drink **Other Milk** such as: whole milk, 2% reduced fat milk, milkshake?

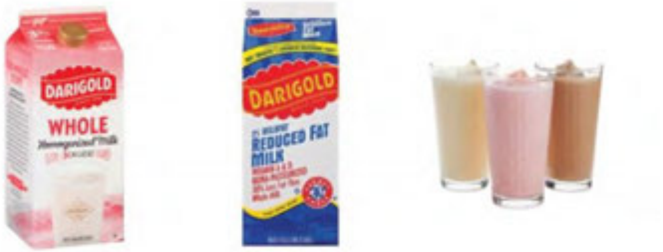

☐ **Never** ☐ **Sometimes** ☐ **Every day** → If every day, more than 1 per day?
☐ **Yes** ☐ **No**

14. How often do you eat **Other Dairy** such as: cheese, eggs, yogurt?

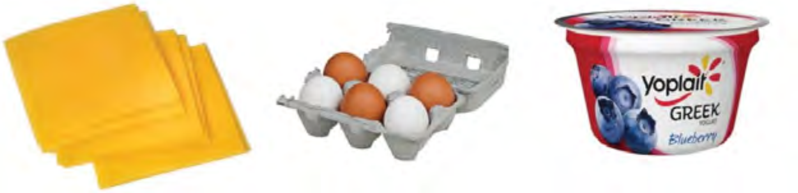

☐ **Never** ☐ **Sometimes** ☐ **Every day** → If every day, more than 1 per day?
☐ **Yes** ☐ **No**

15. How often do you eat **Mixed Dishes** such as: Navajo taco, Navajo burger, burrito?

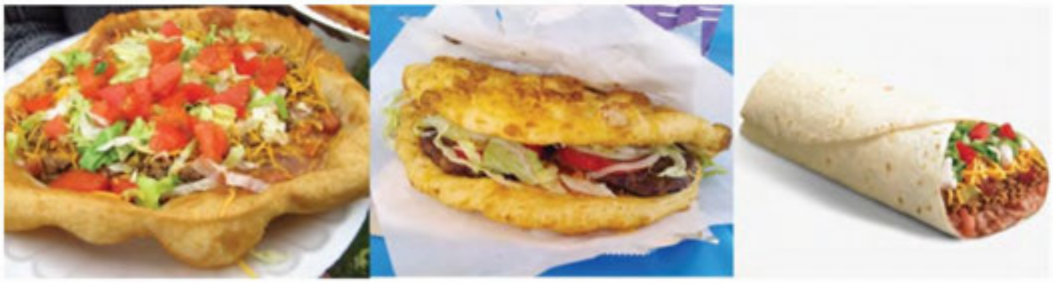

☐ **Never** ☐ **Sometimes** ☐ **Every day** → If every day, more than 1 per day?
☐ **Yes** ☐ **No**

16. How often do you eat **Rice** such as: fried rice, rice?

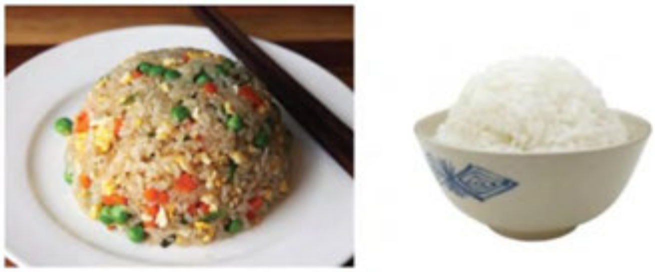

☐ **Never** ☐ **Sometimes** ☐ **Every day** → If every day, more than 1 per day?
☐ **Yes** ☐ **No**

17. How often do you eat **Processed Meat** such as: bologna, salami, lunch meat or processed meat?

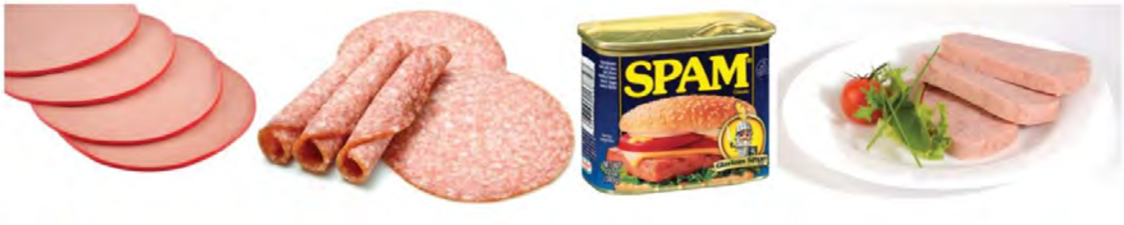

☐ **Never** ☐ **Sometimes** ☐ **Every day** → If every day, more than 1 per day?
☐ **Yes** ☐ **No**

18. How often do you eat **Red Meat** such as: ground beef, meatloaf, pork chops?

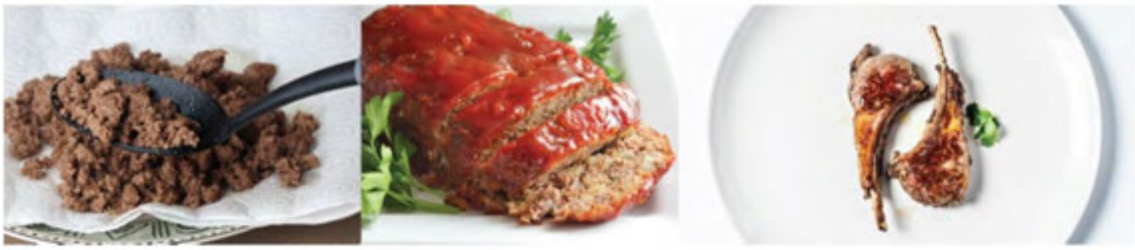

☐ **Never** ☐ **Sometimes** ☐ **Every day** → If every day, more than 1 per day?
☐ **Yes** ☐ **No**

19. How often do you eat **Chicken** such as: any chicken baked, any chicken fried, roasted etc?

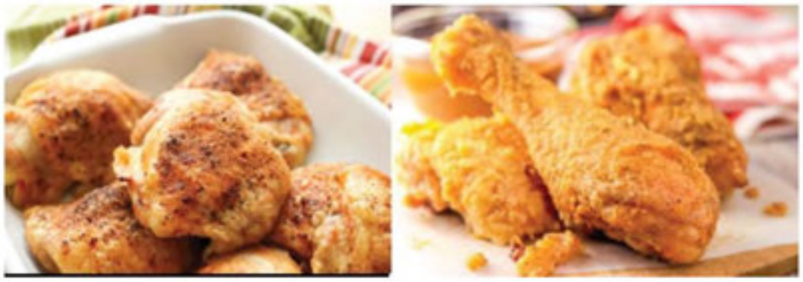

☐ **Never** ☐ **Sometimes** ☐ **Every day** → If every day, more than 1 per day?
☐ **Yes** ☐ **No**

20. How often do you eat **Fish/Seafood** such as: any fish, shrimp, tuna or tuna salad?

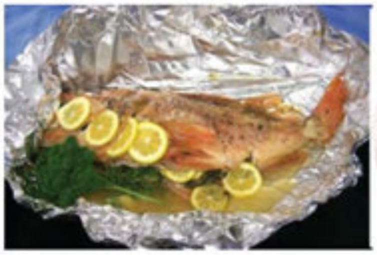
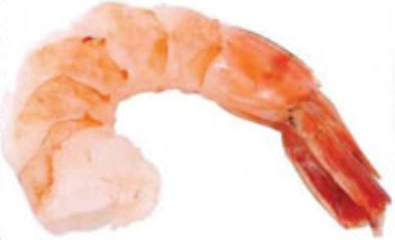
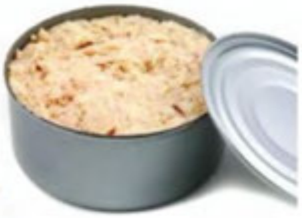

☐ **Never** ☐ **Sometimes** ☐ **Every day** → If every day, more than 1 per day?
☐ **Yes** ☐ **No**

21. How often do you eat **Dessert** such as: ice cream, apple pie, cake?

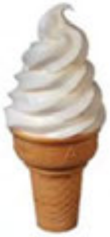
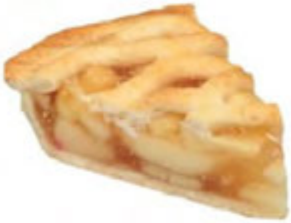
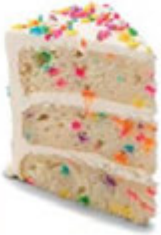

☐ **Never** ☐ **Sometimes** ☐ **Every day** → If every day, more than 1 per day?
☐ **Yes** ☐ **No**

22. How often do you eat **Muffins or Fruit Breads** such as: banana bread, fruit bread or fruit cake, muffin?

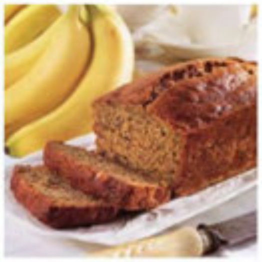
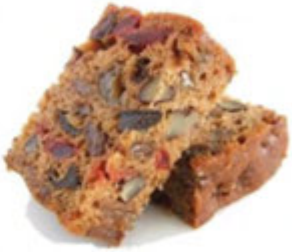
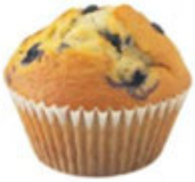

☐ **Never** ☐ **Sometimes** ☐ **Every day** → If every day, more than 1 per day?
☐ **Yes** ☐ **No**

23. How often do you eat **Cookies** such as: cookies, Rice Krispies treat?

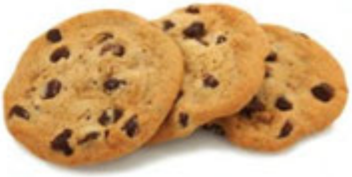
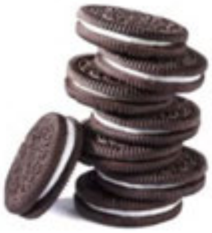
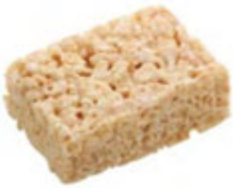

☐ **Never** ☐ **Sometimes** ☐ **Every day** → If every day, more than 1 per day?
☐ **Yes** ☐ **No**

24. How often do you eat **Nuts** such as: nuts, peanut butter?

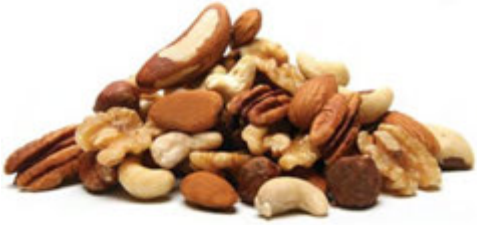
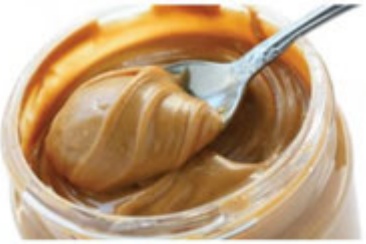

☐ **Never** ☐ **Sometimes** ☐ **Every day** → If every day, more than 1 per day?
☐ **Yes** ☐ **No**

25. How often do you drink **Sweetened Drinks** such as: fruit punch, any regular soft drinks?

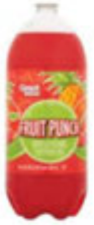

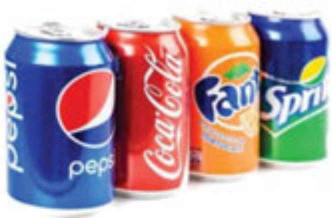

☐ **Never** ☐ **Sometimes** ☐ **Every day** → If every day, more than 1 per day?
☐ **Yes** ☐ **No**

26. How often do you eat **Fruit Juice** such as 100% orange juice, 100% apple juice?

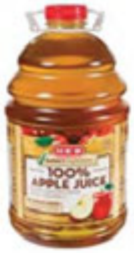

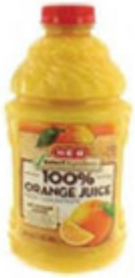

☐ **Never** ☐ **Sometimes** ☐ **Every day** → If every day, more than 1 per day?
☐ **Yes** ☐ **No**

THANK YOU FOR COMPLETING THIS SURVEY!

Supplementary material: Self-efficacy questions [illegible]

SELF-EFFICACY – SECTION 6

The next questions ask whether you think you can or cannot do a certain behavior. I will read each question carefully, and then you will choose one answer. There are no right or wrong answers. (Please check only one answer)

	**Do You Think You Can?**	I know I can	I think I can	I'm not sure I can	I know I can't
27.	I think I can add fruit to my cereal for breakfast.	☐1	☐2	☐3	☐4
28.	I think I can eat a vegetable that's served for lunch.	☐1	☐2	☐3	☐4
29.	I think I can eat a fruit that's served for lunch.	☐1	☐2	☐3	☐4
30.	I think I can bring a fruit to school in my lunch.	☐1	☐2	☐3	☐4
31.	I think I can bring a vegetable to school in my lunch.	☐1	☐2	☐3	☐4
32.	I think I can eat a serving of vegetables for dinner.	☐1	☐2	☐3	☐4
33.	I think I can eat my favorite fruit instead of my usual dessert.	☐1	☐2	☐3	☐4
34.	I think I can help cook a dish with vegetables.	☐1	☐2	☐3	☐4
35.	I think I can help cook a dish with fruit.	☐1	☐2	☐3	☐4
36.	I think I can help shop for fruits and vegetables.	☐1	☐2	☐3	☐4
37.	I think I can read a recipe.	☐1	☐2	☐3	☐4
38.	I think I can use a sharp knife to chop fruits and vegetables.	☐1	☐2	☐3	☐4
39.	I think I can grow fruits or vegetables at my house.	☐1	☐2	☐3	☐4
40.	I think I can grow fruits or vegetables at a school garden.	☐1	☐2	☐3	☐4
